# Postnatal care coverage and its determinants in Sri Lanka: analysis of the 2016 demographic and health survey

**DOI:** 10.1186/s12884-021-03770-0

**Published:** 2021-04-13

**Authors:** Upuli Amaranganie Pushpakumari Perera, Yibeltal Assefa, Uttara Amilani

**Affiliations:** 1 Ministry of Health, Colombo, Sri Lanka; 2 The University of Queensland, Brisbane, Australia

**Keywords:** FPNC, Demographic and health survey, Socio-demographic factors

## Abstract

**Background:**

Postnatal care (PNC) is important for preventing morbidity and mortality in mothers and newborns. Even though its importance is highlighted, PNC received less attention than antenatal care. This study determines the level of PNC coverage and its determinants in Srilanka.

**Methods:**

This is a secondary analysis of the 2016 Demographic and Health Survey. Receiving full postnatal care (FPNC) was defined with a set of indicators to detect adequate care for mother and newborn. Demographic and socio-economic associated factors for receiving FPNC were identified using binary and multiple logistic regression. Variables that had marginal relationship with receiving FPNC which *p*-value less than or equal to 0.2 at binary analysis were selected and included in the multiple logistic regression models. We used manual backward stepwise regression to identify variables which had independent association with receiving FPNC on the basis of adjusted odds ratios (AOR), with 95% confidence interval (CI) and *p*-value less than 0.05. All analyses were performed in SPSS 25.

**Results:**

Of the 8313 women with a live birth in the last 5 years, more than 98% had received postnatal care at facility at least 24 h. More than three-fourth of mothers (*n* = 5104) received the FPNC according to WHO guideline. Four factors were positively associated with receiving FPNC: mothers received antenatal home visits by Public health midwife (AOR = 1.98, 95% CI 1.65–2.39), mothers who got information about antenatal complications and places to go at antenatal clinics (AOR = 1.56, 95% CI 1.27–1.92), been Sinhala (AOR = 1.89, 95% CI 1.35–2.66) and having own mobile phone (AOR = 1.19, 95% CI 1.02–1.38). Mothers who are residing in rural area (AOR = 0.697 95% CI = 0.52–0.93] compared to those who reside in urban areas and maternal age between 20 and 34 years [AOR = 0.72, 95% CI 0.54–0.97] compared to maternal age less than 20 years were detected as negatively associated.

**Conclusion:**

Receiving FPNC in Srilanka is high. However, inequity remains to be a challenge. Socio-demographic factors are associated with FPNC coverage. Strategies that aim to improve postnatal care should target improvement of non-health factors as well.

## Background

The postnatal (PN) period begins immediately after the birth of the baby and extends up to 42 days after birth [1]. It consists of immediate (covers the first 24 h from birth), early (from Days 2 through 7), and late (from Days 8 through 42) periods for the purposes of describing care provision. Close direct or indirect supervision by a skilled attendant is required in the immediate period when the risks are highest More than 60% of global maternal deaths occur during the PN period [1]. According to a review, 45% of postpartum maternal deaths occur within 1 day of delivery; approximately 65% occur within 1 week and roughly 80% occur within 2 weeks [2]. High levels of maternal mortality is detected in all three periods; antepartum, intra-partum and postpartum in Sub-Saharan African (SSA) countries [3]. Globally, 2.4 million neonates died in 2019, around one million within first 24 h of life. The vast majority occurred in lower and middle income countries (LMIC) [4]. Hence, postnatal care (PNC) for the women and the child is important in detecting and treating complications that occur during the delivery as well as providing information for the mother on her and her newborn health. Even though the PNC period is associated with risk, it is the most neglected period for the provision of quality care. Less attention was given for PN period in developing countries [5–8]. Postnatal care utilization in SSA countries was around 52% ranging from 31 to 73% according to DHS survey analysis from 2006 to 2018 in 36 SSA countries [9]. Receiving a PN check ranged from 26 to 94% in SSA countries [5]. Only half of Ugandan postpartum women attended PNC services within 2 days of childbirth [6]. In Myanmar, only around a quarter has received PNC, 4 checkups in postpartum period [7]. According to the most recent country-level data, an average of 62% of women, ranging from 16 to 100%, around the world attends PN visits. PN visits for neonates are on average 36% ranging from 4 to 100% [8]. Even though with the increase in facility delivery, few women and newborn stay in the facility for the recommended 24 h after birth [4].

To help countries improve the situation, the World Health Organization (WHO) provided recommendations on how a mother who has just delivered should be treated. It recommends receiving first PN contact within the immediate PN period. In addition to that, minimum three additional PN checkups are recommended. It further recommends at least 24 h facility care for healthy mother and newborn. Home visits for mother and baby are also recommended [10]. Countries develop their national guidelines for the improvement of PNC based on the WHO’s recommendation [11–13]. In SriLanka, Family Health Bureau (FHB), focal point for maternal and childcare has updated the PNC in 2011 in its maternal care package [11]. It clearly describes how to provide quality hospital and field care for PN mothers and newborns. According to maternal discharging policy in Sri Lanka, immediate PNC during is given at a hospital. Once the mother is discharged from the hospital, professional PNC is provided through home visits by the Public health midwife (PHM) and PN clinics conducted by Medical officers of Health (MOOH) [11].

Maternal and neonatal health (MNH) of SriLanka is a part of the reproductive, maternal, neonatal, child, adolescence and youth healthcare programme, which has been evolved over many decades. Family Health Bureau is the focal point for planning, implementation, monitoring and evaluation of MNH. Services are supplied by preventive and curative health systems at provincial and district level [14]. Sri Lanka has achieved a remarkably better maternal health status despite its weak economic status [15]. Investments into human development such as free education and free health care have contributed significantly to these achievements [16, 17]. More than 99% of pregnant mothers are receiving antenatal care, institutional delivery and immediate PNC in institutions [16]. Quality of PNC received at institutions has been assessed with different studies and found satisfactory according to clients [18, 19]. There is very little information regarding the receiving various aspects of postnatal care for mothers and newborns such as institutional care, time of receiving care, home visit care according to above mentioned guidelines. There is also paucity of research to determine the determinants of receiving PNC among mothers in SriLanka. Therefore the objective of this study is to determine the level of PNC coverage and its determinants in SriLanka.

## Methods

### Data source

We used data from the Sri Lanka Demographic and Health Survey (SLDHS) 2016. Sri Lanka constitutes 25 administrative districts within nine provinces. The SLDHS 2016 covered all 25 districts including the Northern Province, which was excluded in early surveys due to the civil war there [20].

### Sampling

The sampling technique used to select respondents in SLDHS was two- stage stratified cluster sampling design. The sampling frame consisted of census blocks, the subdivision of a Grama niladhari division which is the smallest administration unit at village level. At the first stage, 2500 census blocks were selected as primary sampling units (PSU). At the second stage, 12 housing units were selected from each selected PSU as the secondary sampling unit from all strata except from the strata of the districts in Western Province. In these districts, 10 housing units were selected from each selected PSU. Totally, 28,800 housing units were selected for the survey. The detail of sampling is provided in the freely available SLDHS report [20].

Within the households, 18,510 married women aged 15–49 years were selected for interview and 18,302 interviewed with 98.9% response rate. SLDHS 2016 used computer assisted personal interview first time in the history of the Department of Census and Statistics (DCS) for field data collection. Trained data collecting officers collected detailed data on mothers’ reproductive health behaviours and birth details of children with socioeconomic and demographic data. The dataset obtained from the DCS included 18,302 ever-married women. The current analysis included mothers of 8313 children born between 2011 and 2016. We assessed PNC separately in accordance to WHO guideline and Srilanka guidelines developed by FHB.

#### Outcome variables

Women those who have received full postnatal care (FPNC) in their PN period who delivered between 2011 and 2016 were considered as outcome variable. The outcome variable was recoded as a binary variable (yes/no) in the merged dataset. Receiving FPNC was defined with a set of indicators to detect adequate care for mother and newborn.

Composite score for receiving WHO FPNC was developed using following indicators

1. Had postpartum hospital stay more than 24 h
2. Received PN check-up within 24 h
3. Had additional 3 check-ups within 6 weeks of delivery.
  This additional three may be in field or clinic setup. Therefore two different data; at least three PHM PN home visits irrespective of PN clinic visits or at least two PHM home visits with a PN clinic visit were used to calculate this indicator.

Another composite score for PNC guided by FHB was developed using following indicators which have been collected data in SLDHS 2016.

1. Had postpartum hospital stays more than 24 h
2. Received PN check-up within 24 h
3. Had a checkup by a doctor before discharge
4. Received a home visit by PHM before 5 days after delivery
5. Received at least four PN home visits by PHM within 6 weeks of delivery
6. Attended a PN clinic.

Both composite indicators took the value of 1 if all the above conditions are met.

#### Independent variables

A set of socio-demographic, economic and obstetric care factors which related to the receiving FPNC was used as independent variables. Socio-demographic characteristics were place of residence (urban, rural, estate) including province and district, religion (Buddhist and non-Buddhist) and ethnicity (Sinhala and non-Sinhala), maternal age at delivery(20 to 34 and below 20 / above 35), education status of mother and father (AL / above AL and up to pass OL), occupation status of mother and father (occupied or not), birth order (first born and others) and sex of the child (male or female), involvement of the mother with healthcare decisions and other house-hold decisions, frequency of reading newspapers, listening to radio and watching television of mothers, ownership of mobile phone and bank account of mother and Wealth Index (WI) (richer/ richest and medium/poorer/poorest). Included obstetric care related factors were the details in antenatal care and delivery. Antenatal registration by PHM (yes or no), antenatal clinics attendance (yes or no), antenatal clinic frequency (4 or more and less than 4), antenatal clinic made awareness on complication and services (yes or no), antenatal home visits by PHM (yes or no), place of delivery (institution and non-institution) and mode of delivery (vaginal or other) were used.

#### Measuring socio-economic status

WI and quintile were constructed to represent household economy using the available wealth variables such as housing materials, water source, type of latrines, availability of electricity, and ownership of durable consumer items such as radio, television, mobile phone, land phone, refrigerator, bicycle, motorcycle etc. Principal component and factor analysis statistical method was used to calculate the WI [21].

#### Data analysis

Data were analyzed with SPSS version 25. Basic socio-demographic characteristics of the study sample were presented as descriptive data in frequency and percentages. PN care indicators and prevalence of receiving FPNC according to WHO guideline and FHB guidelines were presented separately as number and percentages.

Associated factors for receiving WHO FPNC were identified using binary and multiple logistic regression (MLR) tests. Variables that had marginal relationship with receiving FPNC which *p*-value less than or equal to 0.2 at binary analysis were selected in to MLR models for controlling the possible effect of confounders. We used manual backward stepwise regression to identify variables which had independent association with receiving WHO FPNC on the basis of adjusted odds ratios (AOR), with 95% CI and *p*-value less than 0.05.

## Results

### Background characteristics

A total of 18,302 ever married women of reproductive age, were included in the SLDHS 2016. Of those, only 8313 women who had their last birth in the 5 years preceding the survey were analyzed here. The overall characteristics of study population are tabulated in Table 1. The majority (78%, *n* = 5523) of the mothers were residing in rural areas, were belong to 20 to 34 years age group (75%, *n* = 5372) and were non-working (77%, *n* = 5535). Most of them were involving healthcare decisions (85%, *n* = 6136) and other household decisions (76.1%, *n* = 5472). Almost all (99.6%, *n* = 8276) were delivered at institutions. Majority had registered with PHM (99%, *n* = 7047), had antenatal clinic visits (99%, *n* = 7118) and received antenatal home visits by PHM (90.6%, *n* = 6510).

**Table 1:** Basic socio-demographic characteristics of mothers’ with children 0–5 years of age, Sri Lanka 2016/2017 (*n* = 8313)

Characteristic	Number	Percentage
**Residence** (*n* = 7077)		
Urban	1114	15.7
Rural	5523	78.0
Estate	440	6.2
**Province**		
Western	1545	18.6
Central	1061	12.8
Southern	980	11.8
Northern	1011	12.2
Eastern	931	11.2
North western	887	10.7
North central	556	6.7
Uva	594	7.1
Sabaragamuwa	748	9.0
**District**		
Colombo	546	6.6
Gampaha	616	7.4
Kalutara	383	4.6
Kandy	528	6.4
Matale	212	2.6
Nuwaraeliya	321	3.9
Galle	387	4.7
Matara	328	3.9
Hambantota	265	3.2
Jaffna	237	2.9
Mannar	213	2.6
Vavuniya	202	2.4
Mulathive	173	2.1
Kilinochchi	186	2.2
Batticaloa	280	3.4
Ampara	401	4.8
Trincomalee	250	3.0
Kurunagala	589	7.1
Puttlam	298	3.6
Anuradhapura	349	4.2
Polonnaruwa	207	2.5
Badulla	326	3.9
Monaragala	268	3.2
Rathnapura	427	5.1
kegalle	321	3.9
	8313	100.0
**Religion** (*n* = 7077)		
Buddhist	4375	61.8
Hindu	1284	18.1
Islam	815	11.5
Roman Catholic	508	7.2
Other Christian	92	1.3
Other	3	.0
**Nationality** (*n* = 7077)		
Sinhala	4648	65.7
Sri Lanka Tamil	1444	20.4
Indian Tamil	215	3.0
Sri Lanka Moor	743	10.5
Muslim	11	0.2
Malay	16	0.2
**Maternal working state** (*n* = 7187)		
Non-working	5535	77.0
Working	1652	23.0
**Father’s working state (*n*** **= 6097)**		
Non-working	109	1.8
Working	5988	98.2
**Maternal education **(*n* = 7187)		
Primary or no schooling	360	5.0
6–10	3165	44.0
Passed OL	1173	16.3
Passed AL	2089	29.1
Degree or higher	400	5.6
**Father’s education** (*n* = 6097)		
Primary or no schooling	499	8.2
6–11	3009	49.4
OL	994	16.3
AL	1338	21.9
Degree or higher	257	4.2
**Mother’s age at delivery** (years) *n* = 7140		
15–19	460	6.4
20–34	5372	75.2
35–49	1308	18.3
**Household wealth index (*n* = 7077)**		
Poorest	1760	24.9
Poorer	1463	20.7
Middle	1362	19.2
Richer	1362	19.2
Richest	1130	16.0
**Healthcare decisions making** (*n* = 7187)		
Mother involved	6136	85.4
Mother not involved	1051	14.6
**Other household decision making** (*n* = 7187)		
Mother involved	5472	76.1
Mother not involved	1715	23.9
**Having access to any media frequently (*n*** **= 6869)**		
Yes	6104	88.9
No	765	11.1
**Birth order**		
First born	3214	38.7
Second or Third	4581	55.1
Fourth or fifth	475	5.7
Six or more	43	0.5
**Sex of child**		
Male	4279	51.5
Female	4034	48.5
**Age of child (years)**		
0–1	1500	18.2
1–2	1560	19.0
2–3	1708	20.8
3–4	1673	20.4
4–5	1709	20.8
5+	71	0.9
**Place of birth**		
Government hospital	7880	94.8
Private hospital	394	4.7
Other	39	0.5
**Place of birth**		
Institutional	8276	99.6
Non-institutional	37	0.4
**Mode of delivery**		
Normal vaginal delivery	5728	68.9
Caesarean section	2461	29.6
Other	85	1.0
**Antenatal care (*n* = 7187)**		
Received	7118	99.0
Not	69	1.0
**Registered by** ^a^**PHM (*n* = 7118)**		
Yes	7047	99.0
No	71	1.0
**Antenatal clinic frequency (*n* = 7118)**		
More than or equal four visits	7078	99.4
Less than four visits	40	0.6
**Antenatal tetanus toxoid (*n*** **= 7072)**		
Yes	6831	96.6
No	241	3.4
**Antenatal awareness on where to go with complications (*n*** **= 7094)**		
Yes	6540	92.2
No	554	7.8
**Antenatal home visits by** ^a^**PHM**(*n* = 7187)		
Yes	6510	90.6
No	677	9.4
**Number of antenatal home visits by** ^a^**PHM (*n* = 6510)**		
1	587	9.0
2	854	13.1
3	1300	20.0
> =4	3770	57.9

^a^*PHM* public health midwife

### Postnatal care coverage

Vast majority (> 98%) of postnatal mothers had received postnatal care at facility at least 24 h. More than 95% of mothers received home care by PHM. Relatively low coverage were detected in home visit by PHM within 5 days (59%, *n* = 3935) and receiving at least four postpartum home visits by PHM (26%, *n* = 1712). Around 78% (*n* = 5606) of mothers attended postnatal clinic conducted by MOOH, and less than 80% (*n* = 4410) of them were examined by a doctor (Table 2).

**Table 2:** Post-partum care indicators among mothers with children less than 5 years of age, Sri Lanka, 2016/2017

	Indicator	Sample size	Number	Percentage %
1	Received Postnatal care at facility at least 24 h	8260	8118	98.3
2	Received Postnatal check-up within one hour of delivery	7647	6125	80.1
3	Received Postnatal check-up within 4 h of delivery	7526	7325	97.3
4	Received Postnatal check-up within 24 h of delivery	7647	7526	98.4
5	Received Postnatal check-up by a skilled personal	7647	7607	99.5
6	Received Postnatal check-up before discharge by a doctor	7988	7855	98.3
7	Received post-partum home visits by PMH	8313	7933	95.4
8	Received first post-partum home visit by PHM within 5 days of delivery	6646	3935	59.2
9	Received first post-partum home visit by PHM within 10 days of delivery	6646	5687	85.6
10	Received at least 4 post-partum home visits by PHM	6595	1712	26.0
11	Received at least 3 post-partum home visits by PHM	6595	3712	56.3
12	Received at least 2 post-partum home visits by PHM	6595	5546	84.1
13	PHM has made aware of mothers at home visits regarding the available services in postnatal care	6757	6333	93.7
14	Attended Postnatal clinic conducted by MOOH	7187	5606	78.0
15	Postnatal mother had a check by doctor at the postnatal clinic	5606	4410	78.7
16	Postnatal baby had a check by doctor at the postnatal clinic	5606	5399	96.3
17	Received additional 3 check-ups within 6 weeks of delivery: in the field or clinic setup.			
18	Received FPNC according to WHO guideline	6641	5104	76.9
19	Received FPNC according to FHB guideline.	6220	860	13.8
20	Received field related FPNC care according to FHB guideline.	6468	926	14.3
21	Received within the institution FPNC care according to FHB guideline.	7634	7264	95.2

*PHM* public health midwife, *MOOH* Medical officers of health, *FPNC* full postnatal care, *FHB* Family Health Bureau

More than three fourth of mothers (76.9%, *n* = 5104) received the FPNC according to WHO guideline while only 13.8% (*n* = 860) received FPNC according to FHB guideline. FPNC received within the institution was 95.2% (*n* = 7264) while the field related care part was 14.3% (*n* = 926) according to the FHB guidelines (Table 2).

### Determinants of receiving FPNC

Determinants of receiving FPNC according to WHO guideline were presented in Tables 3 and 4 as Crude OR and AOR respectively. In MLR model, mothers who received antenatal home visits by PHM were 1.99 (AOR = 1.99, 95% CI 1.65–2.39, *P* < 0.001) times more likely to receive FPNC than those without antenatal PHM home visits. Similarly, mothers who got information about antenatal complications and places to go at antenatal clinic (ANC) were 1.56 times (AOR = 1.56, 95% CI 1.27–1.92, P < 0.001) more likely to receive FPNC than who did not. Mothers ethnicity, been Sinhala, major nationality in SriLanka (AOR = 1.89, 96% CI 1.35–2.66, *P* < 0.001) and, having own mobile phone (AOR = 1.19, 95% CI 1.02–1.38, *P* = 0.027) were other factors which are significantly associated with receiving FPNC.

**Table 3:** Association of receiving full postnatal care according to WHO guidelines with socio-demographic and obstetric care related factors

Variable	Receiving FPNC WHO				
	No	%	Crude OR	95%CI	***P*** value
**Socio-demographic factors**					
**Residence** (*n* = 6542)^a^					
Rural	4021	78.1	1.337	1.168–1.530	0.000
Urban+ Estate	1015	72.8			
**Religion** (*n* = 6542)^a^					
Buddhist	3270	80.4	1.652	1.470–1.856	0.000
Non-Buddhist	1766	71.3			
**Nationality** (n = 6542)^a^					
Sinhala	3473	80.5	1.762	1.566–1.982	0.000
Non-Sinhala	1563	70.1			
**Mother’s age at delivery** (years) (*n* = 6600)^b^					
20–34	1257	77.5	1.047	0.916–1.197	0.497
Below 20 and above 35	3817	76.7			
**Maternal working state** (*n* = 6641)^c^					
Working	1184	77.7	1.063	0.927–1.219	0.379
Non-working	3920	76.6			
**Father’s working status** (*n* = 5649)^d^					
Working	4276	77.1	1.289	0.830–2.002	0.256
Non-working	73	72.3			
**Maternal education** (*n* = 6641)^c^					
AL and above	1815	78.5	1.158	1.026–1.308	0.018
Up to passed OL	3289	76.0			
**Father’s education** (*n* = 5649)^d^					
AL and above	1147	77.1	1.007	0.875–1.160	0.918
Up to passed OL	3202	77.0			
**Household wealth index quintile (*n* = 6542)**^e^					
Richer/ Richest	1824	79.0	1.199	1.061–1.355	0.004
Poorest/ Poorer/ Middle	3212	75.9			
**Healthcare decisions making ability** (*n* = 6641)^c^					
Mother involved	4373	77.0	1.058	0.901–1.242	0.490
Mother not involved	731	76.0			
**Other household decision making ability (*n* = 6641)**^c^					
Mother involved	3916	77.3	1.121	0.982–1.279	0.090
Mother not involved	1188	75.3			
**Spending decision on father’s earning (*n*-6641)**^c^					
Mother involved	3564	77.4	1.096	0.970–1.239	0.142
Mother not involved	1540	75.7			
**Maternal access to any media frequently (*n*** **= 6363)**^f^					
Yes	4361	77.0	0.982	0.815–1.185	0.852
No	543	77.4			
**Newspaper read by mother frequently (*n*** **= 6363)**^g^					
Yes	1994	76.5	0.946	0.840–1.065	0.356
No	2916	77.5			
**Watch TV by mother frequently (*n*** **= 6635**)^h^					
Yes	4098	76.9	1.018	0.883–1.175	0.802
No	1001	76.6			
**Listening radio frequently(*n*** **= 6641)**^i^					
Yes	2455	77.1	1.030	0.919–1.154	0.613
No	2649	76.6			
**Mother own mobile phone (*n* = 6641)**^i^					
Yes	4096	77.6	1.225	1.068–1.405	0.004
No	1008	73.9			
**Mother own bank account (***n*** = 6641)**^i^					
Yes	4242	77.6	1.258	1.090–1.454	0.002
No	862	73.4			
**Mother use internet ever(***n*** = 6641)**^i^					
Yes	1033	76.0	0.943	0.819–1.084	0.408
No	4071	77.1			
**Obstetric care related factors**					
**Antenatal Registration by PHM (*n*** **= 6587)**^j^					
Yes	5018	76.9	0.901	0.487–1.668	0.740
No	48	78.7			
**Antenatal clinic care (*n* = 6641)**^k^					
Received	5066	76.9	1.402	0.780–2.522	0.257
Not received	38	70.4			
**Antenatal clinic made awareness on complication and services (*n*** **= 6573)**^l^					
Yes	4736	77.9	1.917	1.579–2.329	< 0.001
No	320	64.8			
**Antenatal clinic attendance frequency (***n*** = 6587)**^m^					
4 or more	5046	77.0	2.344	1.181–4.652	0.012
Less than 4	20	58.8			
**Antenatal tetanus toxoid (*n*** **= 6552)**^n^					
Received	4882	77.1	1.169	0.857–1.593	0.323
Not received	161	74.2			
**Antenatal home visits by PHM** (*n* = 6641)^o^					
Yes	4716	78.1	1.966	1.646–2.348	< 0.001
No	388	64.5			
**Place of birth**					
Institution	5104	76.9	Not calculated		
Non-institution	0	0			
**Mode of delivery (*n* = 6641)**^o^					
Normal vaginal delivery	3516	76.0	0.852	0.751–0.966	0.013
Other	1588	78.8			
**Birth order (*n* = 6641)**^o^					
First born	1799	75.9	0.923	0.820–1.039	0.187
Others	3305	77.4			
**Sex of child (*n* = 6641)**^o^					
Male	2650	77.9	1.124	1.003–1.260	0.044
Female	2454	75.8			

*FPNC* full postnatal care, *PHM* Public health midwife

a-o = missing values, ^a^506; ^b^1526; ^c^1537; ^d^23; ^e^606; ^f^1459; ^g^1453; ^h^1536; ^i^1537; ^j^1521; ^k^1537; ^l^1517; ^m^1521; ^n^1509; ^o^1537

**Table 4:** Association of receiving full postnatal care according to WHO guidelines with socio-demographic and obstetric care related factors

	Variable	AOR	95% CI	***P*** value
1	Residence			
	Rural	0.697	0.522–0.933	0.015
	Estate	0.830	0.639–1.078	0.163
2	Religion: Buddhist	0.853	0.610–1.193	0.353
3	Nationality: Sinhala	1.892	1.346–2.662	< 0.001
4	Mother’s age at delivery (years)			
	20–34	0.724	0.541–0.968	0.029
	Above 35	0.851	0.721–1.003	0.054
5	Maternal education: AL or above	1.006	0.873–1.158	0.936
6	Household wealth index quintile	1.069	0.925–1.237	0.367
7	Mothers involve in household decision making ability	1.041	0.898–1.207	0.590
8	Mothers involve in spending decision on father’s earning	1.039	0.906–1.102	0.586
9	Mother own mobile phone	1.188	1.020–1.384	0.027
10	Mother own bank account	1.037	0.884–1.217	0.655
11	Antenatal clinic made awareness on complication and services	1.562	1.273–1.916	< 0.001
12	Antenatal clinic attendance frequency 4 or more	1.635	0.792–3.376	0.184
13	Antenatal home visits by PHM^*^	1.985	1.648–2.392	0.000
14	Normal vaginal delivery	0.924	0.808–1.056	0.245
15	First born child	0.955	0.834–1.093	0.505

*PHM public health midwife

Mothers who are residing in rural area were 0.7 [AOR = 0.697, 95% CI = 0.52–0.93, *P* = 0.015] times less likely to receive FPNC than those who reside in urban areas. Similarly, maternal age between 20 and 34 years were 0.72 less likely to have FPNC [AOR = 0.72, 95% CI 0.54–0.97, *P* = 0.029] than mothers age less than 20 years. Maternal age with more than 35 years old were 0.85 times [AOR = 0.85, 95% CI 0.72–1.003, *P* = 0.054] less likely to receive FPNC than those with less than 20 even though it was marginally significant.

Mother’s socio-demographic factors such as being Buddhist (OR = 1.65, 95% CI =1.47–1.86) than being Non Buddhist, higher maternal education with Advanced level and above (OR = 1.16, 95% CI =1.03–1.31) than Ordinary level and below, household WI being in richer and richest (OR = 1.19, 95% CI =1.06–1.36) than, poorest, poorer and middle and mothers having own bank account (OR = 1.26, 95% CI = 1.09–1.45) than not having showed positive association in univariate analysis. Likewise having more than 4 ANC visits (OR = 2.34, 95% CI =1.18–4.65) showed positive and normal vaginal delivery (OR = 0.85, 95% CI =0.75–0.97) showed negative association in univariate analysis.

## Discussion

The current analysis of SLDHS 2016 data revealed that nearly all mothers received timely PNC in an institution. More than three fourth (76.9%) of postnatal mothers have received FPNC as recommended by WHO. Results from the MLR analysis indicate the following factors as positively associated with receiving FPNC. Receiving antenatal home visits by PHM, receiving knowledge about antenatal complications and available services in ANC, mothers having own mobile phone and belongs to Sinhala nationality. Likewise, it was found that being in rural area and older maternal age were negatively associated with receiving FPNC.

Almost all mothers receiving timely PNC in an institution reflects the achievements of maternity care in Sri Lanka within the existing free health services. In the field, majority received PHM home visits. Postnatal care indicators recommended by FHB, which related to field activities such as PN home visits within 5 days of delivery and at least four home visits in PN period noticed relatively low values here. They were compatible with data derived from the field [14, 22, 23].

In Sri Lanka, PN home visits are done by highly trained PHMM. Lack of adequate number of competent healthcare workers, especially PHM, who is the grass root level caretaker of mothers and children in the field, may be the reason for receiving less than recommended home visits. Recommended number of PHMM, which is one per 3000 population hardly achieved in highly populated areas [24]. There are large numbers of vacancies in difficult geographical areas. Carrier as PHM in SriLanka becoming unpopulated among younger generation due to perceived hardship of field works comparing to other sophisticated job opportunities for similar education qualifications.

The reasons for low coverage may be due to some maternal factors such as unavailability at home in PN period. Sri Lankan women, especially primi-mothers, prefer to go to their parents after delivery [25, 26]. In such a situation, the care should be given from the second area PHM. There may be communication gaps. Excessive workload of PHM may be associated with low coverage [27]. Further analysis is needed to understand the factors that contribute to low coverage of the field related PNC.

The finding that more than three fourth of PN mothers have received FPNC as recommended by WHO was slightly higher compared to the reports from other countries in the South East Asian region. Nepal DHS 2016 analysis got PN check-up within 7 days of delivery as 54.5% [28]. Complete utilization of PNC in Nepal is around 22%, with the definition of three PNC within 7 days of delivery [29]. Three PN visits were less as 17.5% in another study from Nepal, Chitwan [30]. Similarly a study conducted in Bangladesh, taken PNC more than three times in PP period was 29% [31]. Sri Lankan healthcare system may be more advanced than other regional countries. Socio-demographic variation between different countries as well as within the same country may be another reason for the difference.

Countries in African region showed relatively less coverage. In Nigeria, being the definition of FNAC same, it was 74% in urban areas and 61% in rural areas [32]. In Ethiopia, it was differing as 28% [33] (only three contacts in PP period), 57.5% [34] and 67% [35] with different study settings and different definitions of postnatal care coverage. In SSA countries, the median percentage of women who reported receiving a PN check was 71.7%, ranging from 26.6% in Swaziland to 94.4% in Burkina Faso [5]. In Malawi, 48% PN women had a check by skilled healthcare worker [36]. These differences may be due to the variation of socio-demographic factors as well as time gap of studies. There may be more improvement in MNH services with modern technology and facilities in recent developments in all countries.

The above findings of associated factors show a close linkage between some maternal characteristics, antenatal care and PNC. Getting antenatal home visits by PHM has increased receiving FPNC among mothers. This finding was in agreement with a study from India, which detected that contacts with health worker during pregnancy increased utilization on postnatal care [34]. The fact that the mother was in close relationship with PHM who had home visits would explain the above finding. In antenatal home visits, PHM assesses mother’s health as well as other social determinants of health. She gets an opportunity to provide overall knowledge that improve attitude and practices individually to mothers in home visits [11].

In our analysis, we detected that mothers receiving knowledge about antenatal complications and available services for complications from ANC had received more FPNC. However, the finding was based on a single question used in SLDHS regarding awareness on antenatal complications. Limenehi has found that awareness about maternal complication improves PNC [37]. Receiving antenatal care showed positive association with receiving PNC in several other studies. Uptake of recommended number of ANC visits was a factor which increases PNC [6, 36, 38, 39]. A timely first ANC visit and receiving the adequate number of tetanus injections showed association with PNC as detected by Khaki [36]. Not only are the visits, the content of care received during ANC detected as important [39]. Even though some studies elicited a positive association between, receiving recommended number of antenatal clinic visits and PNC, we were unable to elicit it here [6, 36, 38, 39]. Similarly, several studies found that content of care received and components of ANC were associated with PNC attendance [40]. Here we could not elicit it due to the fact that almost all ANC related components have achieved coverage of more than 99%.

Our analysis found that mothers with own mobile phones received more PNC. This could be due to women who have mobile phones may have more autonomy and can efficiently contact health staff. The results are consistence with findings that women’s phone ownership and usage is generally associated with better reproductive care indicators [41, 42]. Using m-health interventions to strengthen postnatal care has proven benefits [43, 44]. The assessment here was only covers one question in SLDHS regarding the ownership of a mobile phone, which may need further information on usage for further inferences.

The current analysis shows that mothers from rural areas are less likely to receive FPNC compared to mothers in urban areas. This is similar to what was reported in other studies in Ethiopia [35, 38] Uganda [6], Malawi [36] and Indonesia [45] as high postnatal access in urban mothers. Many factors may contribute to the results. Health care facilities are more concentrated in urban areas [46]. More educated and financially stable women may live in urban areas, which may enhance the receiving healthcare. Contradictory finding detected in Nepal DHS 2016 as urban area mothers have less PNC [28].

Our finding, that older maternal age was associated with less FPNC, is tallying with Rwanda mothers [47]. Older women may have thought that PNC as not necessary with their previous experiences. Younger women less than 20 years of age are considered as high risk due to teenage years and have drawn more attention in field and hospital level in SriLanka. This may be an explanation for the above result. In contrast to our findings, Khaki detected older age as receiving more PNC in Malawian women [36].

The other factor which detected strong positive association in several studies around the globe was the institutional delivery [6, 28, 34, 48]. We could not demonstrate that association due to the fact that more than 99% deliveries occurred in institutions. Mode of delivery being caesarian received more PNC, which could not be detected with our analysis [5, 33, 36, 38].

The main strength of this study is that it is based on a nationally representative sample. SLDHS collect quality data with rigorous methodology with the involvement of experienced staff. 2016 survey included all nine provinces in the country including Northern Province which was excluded in previous surveys due to conflict situation there. Data were collected tallying with pregnancy record whenever available. Therefore, the recall bias may be minimized. The major limitation is the cross-sectional nature of the SLDHS survey, which means that only associations could be detected between the variables, not causality. Another limitation is that due to secondary data, some important associated variables may have missed such as family support, distance to health facilities, availability of healthcare personals, etc. Actual figure of receiving FPNC may be higher than the analyzed one. SLDHS 2016 has not collected data on private sector contacts in PN period. It collected data on PN clinic visit conducted by MOOH only. Private checkups with a consultant or a general practitioner are prevailing in some areas of the country. The study has included mothers who gave birth on last 5 years. Therefore there might be a possibility of some recall bias.

## Conclusion

Almost all PN mothers in Sri Lanka received institutional PN care for more than 24 h. The coverage of receiving FPNC was found to be higher than other countries in the region. Nevertheless, the distribution of receiving care among different socio-economical groups are unequal. Some vulnerable groups such as mothers reside in rural areas; mothers with older maternal age in the country are at risk of being left behind. Healthcare decision makers should target these groups with different strategies than normal routine. Inequality in receiving care among women of different places of residence need to be considered at the policy making level. Future programme efforts should focus on ensuring the equal distribution of resources to promote the equity of care. Strategies that aim to improve PNC should target improvement of non-health factors.

## Data Availability

2016 Sri Lanka Demographic Health Survey dataset is not in the public domain and is a property of the Department of Census and Statistics-Sri Lanka. Additional information and the dataset can be obtained from the Department of Census and Statistics, “Sankyana Mandiraya”, 306/71, Polduwa Road, Battaramulla, Sri Lanka, Telephone: + 94 11 2147000 and + 94 11 2147400, Email: information@statistics.gov.lk,

## Abbreviations

ANC: Antenatal clinic
AOR: Adjusted odds ratios
CI: Confidence interval
DCS: Department of census and statistics
FHB: Family Health Bureau
FPNC: Full postnatal care
LMIC: Lower and middle income countries
MCH: Maternal and child health
MLR: Multiple logistic regression
MNH: Maternal and neonatal health
MOOH: Medical officers of Health
OR: Odds ratios
PHM: Public health midwife
PN: Postnatal
PNC: Postnatal care
PSU: Primary sampling units
SLDHS: Sri Lanka Demographic and Health Survey
SPSS: Statistical Package for Social Sciences
SSA: Sub-Saharan Africa
WHO: World Health Organization
WI: Wealth Index
